# Multi-site binding of epigallocatechin gallate to human serum albumin measured by NMR and isothermal titration calorimetry

**DOI:** 10.1042/BSR20170209

**Published:** 2017-05-11

**Authors:** Joshua D. Eaton, Mike P. Williamson

**Affiliations:** Department of Molecular Biology and Biotechnology, University of Sheffield, Firth Court, Western Bank, Sheffield S10 2TN, U.K.

**Keywords:** bioavailability, catechin, EGCG, ITC, NMR spectroscopy, singular value decomposition

## Abstract

The affinity of epigallocatechin gallate (EGCG) for human serum albumin (HSA) was measured in physiological conditions using NMR and isothermal titration calorimetry (ITC). NMR estimated the *K*_a_ (self-dissociation constant) of EGCG as 50 mM. NMR showed two binding events: strong (*n*_1_=1.8 ± 0.2; *K*_d1_ =19 ± 12 μM) and weak (*n*_2_∼20; *K*_d2_ =40 ± 20 mM). ITC also showed two binding events: strong (*n*_1_=2.5 ± 0.03; *K*_d1_ =21.6 ± 4.0 μM) and weak (*n*_2_=9 ± 1; *K*_d2_ =22 ± 4 mM). The two techniques are consistent, with an unexpectedly high number of bound EGCG. The strong binding is consistent with binding in the two Sudlow pockets. These results imply that almost all EGCG is transported in the blood bound to albumin and explains the wide tissue distribution and chemical stability of EGCG *in vivo*.

## Introduction

(−)−Epigallocatechin gallate (EGCG) is a non-hydrolysable tannin or polyphenol, found in particularly high concentrations in green tea (270 mg l^−1^). Approximately 2 h after drinking a cup of green tea, the EGCG serum concentration reaches a peak level of approximately 0.2 μM [1,2]. In mice, a second dose of EGCG 6 h later increased tissue concentrations by a factor of 4–6 above that found for a single dose, suggesting that drinking green tea throughout the day could result in significantly higher concentrations than this [3]. EGCG has been claimed to have a remarkably wide range of beneficial effects, including as an antioxidant and reducing the risk of cancer, amyloid disease, bacterial infection, HIV infectivity and cardiovascular risk [4,5]. *In vitro*, EGCG in solution is oxidized over a timescale of hours. However *in vivo*, its stability appears to be greater, and it has been suggested that this is because it is bound to human serum albumin (HSA), which protects it [6–8]. HSA is the most abundant protein in human blood and functions as a carrier of various hydrophobic compounds and as a pH and metal ion buffer. HSA has three related domains. Two of these have binding sites for hydrophobic molecules: a pocket on domain IIA known as Sudlow site I, which has the only tryptophan in HSA close to it; and one on domain IIIA known as Sudlow site II.

There have been a number of studies on binding of EGCG and related tannins to either HSA or its closely related bovine homologue, BSA. Studies using fluorescence quenching of the single tryptophan found dissociation constants (*K*_d_) to HSA of 17 μM [9] and 12.5 μM [10], though such studies can only monitor binding close to Sudlow site I and any other binding will be invisible. A study using quartz crystal microbalance found a *K*_d_ of 4 μM [11]. Two molecular docking studies of EGCG/BSA binding suggested binding near Sudlow site I [8,12], while another fluorescence study backed by modelling [9] suggested that EGCG binds HSA at a single site, probably at Sudlow site I. A study of EGCG/BSA binding by affinity capillary electrophoresis found an affinity of 9 μM [13], while studies using tryptophan fluorescence found 0.7 μM [12] and 2.2 μM [8]. Previous studies have therefore measured affinities covering a wide range, between 0.7 and 17 μM, with the consensus at the stronger end. Binding has also been studied using CD (which suggested binding at both Sudlow sites) [14], EPR [15], affinity chromatography [16] and PAGE [7]. Studies of binding of tannins to BSA using isothermal titration calorimetry (ITC) concluded that they bind to two sets of multiple binding sites [17,18]. Dimeric ellagitannins (similar to EGCG but larger and less flexible) were found to bind strongly with *n∼*2 (i.e. two molecules of tannin bound to one BSA) and a *K*_d_ of approximately 20 μM and more weakly with n*∼*20 and *K*_d_*∼*1 mM [17]. The possibility of multiple binding in this last study raises a number of questions about the total number of molecules of EGCG that can be transported by albumin, and prompted our investigation using two complementary techniques: ITC and NMR, the latter of which has been used extensively to probe tannin/peptide interactions, but not interactions with albumin so far.

ITC measures the heat of binding of a ligand (together with any other heat changes when a ligand is injected into a protein solution), while NMR measures changes in chemical shift at specific sites in response to addition of ligand. Each technique has its own problems and benefits. ITC and NMR provided similar and to some extent complementary information, thereby increasing our confidence in the conclusions.

## Materials and methods

### Materials

HSA was obtained as a serum purified lyophilized powder, fatty acid and globulin-free, from Sigma–Aldrich. EGCG was also obtained from Sigma. All ITC, and the self-association study, was carried out in 50 mM phosphate buffer at pH 7.30 ± 0.01 containing 22 mM KH_2_PO_4_, 28 mM Na_2_HPO_4_, 80 mM NaCl and 0.02% sodium azide. HSA was dialysed twice against this buffer. SDS/PAGE gels and NMR of the protein indicated high purity. HSA concentration was measured using an extinction coefficient ε =36500 M^−1^ cm^−1^ [19], and EGCG concentration was measured by NMR against an internal standard of 3-(trimethylsilyl)-2,2′,3,3′-d_4_ propionate (TSP) using a long relaxation delay for complete relaxation. HPLC ESI mass spectrometry (HPLC-ESI-MS) was performed on a maXis ultra-high resolution TOF instrument (Bruker). ITC and NMR self-association data were fitted using a Levenberg–Marquardt non-linear least squares fitting algorithm [20].

### NMR

Spectra were measured on a Bruker Avance-I 800 MHz spectrometer. For the self-association, chemical shifts were measured relative to TSP during dilution of EGCG by phosphate buffer. In order to avoid any effects due to interaction of TSP with EGCG, the TSP was kept in a capillary tube inside a standard 5 mm NMR tube. Solutions were in 90% H_2_O/10% D_2_O and water suppression was by excitation sculpting [21]. Self-association was modelled using two different models. The isodesmic model assumes that the molecule can associate into dimers and then into stacks of higher multimers, and that the affinity for each binding step is the same, given in (eqn 1) [22]:

1 $$\begin{array}{l} \left(\delta_{\mathrm{observed}}-\delta_{A}\right)=\\ (\delta_{\max}-\delta_{A})\;K_{a}\;[A_{0}]{\left\{\frac{2}{\left[1+{\left(4K_{a}[A_{0}]+1\right)}^{1/2}\right]}\right\}}^{2}\;\times \;\left\{2-K_{a\;}[A_{0}]{\left\{\frac{2}{\left[1+{\left(4K_{a}\;[A_{0}]+1\right)}^{1/2}\right]}\right\}}^{2}\right\} \end{array}$$

where* δ*_A_ is the chemical shift of the monomer, *δ*_max_ is the shift in an infinite stack, [A_0_] is the total solute concentration and *K*_a_ is the self-dissociation constant. The modified isodesmic model is the same except that it is assumed that the chemical shift change for a molecule at the end of a stack is only half of the one in the middle of the stack and gives (eqn 2) [23]:

2 $$(\delta_{\mathrm{observed}}-\delta_{A})=(\delta_{\max}-\delta_{A})K_{a}[A_{0}]{\left\{\frac{2}{\left[1+{\left(4K_{a}[A_{0}]+1\right)}^{1/2}\right]}\right\}}^{2}$$

For the titration of EGCG into HSA, a higher concentration of phosphate was required in order to keep the pH constant. It was carried out in 120 mM phosphate, 32 mM NaCl, pH 7.51 ± 0.05. It was also done at 25°C to reduce the amount of protein precipitated during NMR and pH measurements. A solution of HSA was prepared in 500 μl phosphate buffer with pH 7.51, lyophilized and redissolved in the same volume of D_2_O, to keep the same effective pH. Solutions were left for at least 48 h before use, to ensure complete isotope exchange of all NH. The protein concentration was measured as 510 μM. A 5 mM solution of EGCG was prepared in the same deuterated buffer. The pH was checked at every other addition of EGCG and altered wherever necessary to keep the pH within 0.05 pH units. For more accurate measurement of chemical shifts, NMR signals were resolution enhanced by Gaussian multiplication. Chemical shifts were initially measured using either internal TSP or TSP in a capillary, but were observed to change in unexpected ways, possibly as a consequence of changes to bulk susceptibility. Chemical shifts were therefore aligned on a set of aliphatic protein signals that showed no relative change and were assumed to be unaffected by EGCG binding. Binding affinity at each equivalent set of sites was analysed by measuring protein chemical shifts using (eqn 3) [23,24]:

3 $$\Delta \delta_{\mathrm{obs}\;}=\left(\frac{\Delta \delta_{max}}{2n[P_{i}]}\right)\left\{[n\;{\left[P\right]}_{i\;}+{\left[L\right]}_{i\;}+K_{d}] -\sqrt{\left({\left(n{\left[P\right]}_{i}+{\left[L\right]}_{i}+K_{d}\right)}^{2}-4n{\left[P\right]}_{i\;}{\left[L\right]}_{i}\right)}\right\}$$

where *n* is the number of equivalent sites, [P]_i_ and [L]_i_ are the total concentrations of protein and ligand and* K*_d_ is the dissociation constant. For modelling independent binding at two sites, two independent versions were added together. Fittings were calculated using the Solver function in Microsoft Excel. Chemical shift data were processed by SVD in Matlab™. Data were input as a matrix, singular value decomposition was carried out by factorizing the data matrix **D** as **D** = **UWV**^T^, where **W** is the diagonal matrix of singular values, and then all except the first three columns of **U** and **V** and the first three rows and columns of **W** were set to zero. A reduced-noise version of **D** was then recalculated using the new **U, W** and **V** matrices.

### ITC

ITC was carried out on a TA Instruments Low Volume Nano-ITC instrument. The 190 μl sample cell is lined with 24-carat gold and the platinum 50 μl syringe is loaded with titrate. Between each use, the syringe, loading needle and sample cell were cleaned with >50 ml Decon90 (Decon®) followed by 50% ethanol and finally washed with an excess of milli-Q water until the run-through became clear. All solutions were in the phosphate buffer at pH 7.3 and all the solutions were thoroughly degassed for 10 min prior to loading. The syringe contained 50 μl of 5 mM EGCG and the sample cell was overloaded with 300 μl of 200 μM HSA. The reference cell was changed between experimental days or after two titrations, whichever was shorter and contained 300 μl of the same degassed phosphate buffer. Unless stated otherwise, all titrations were carried out at 37°C. After loading, the syringe was set to a stir-rate of 280 rpm and the system was left to reach thermal equilibrium before the titration started, i.e. when background temperature fluctuations reached a gradient of <0.1 μW h^−1^ and a peak-to-peak standard deviation ≤±0.01 μW [25]. The interval time between injections was 300 s with injection volumes of 1.49 μl, apart from the first injection whose volume was set to 0.06 μl. The sample cell volume has previously been determined to be 168 μl by chemical calibration of the instrument using heats of Tris/HCl protonation. Experimental data were exported from the proprietary NanoAnalyze software.

ITC data were processed initially using the NanoAnalyze software and the single independent sites model. However, this model proved inadequate, and the software was unable to deal with two sets of independent sites, and so data were processed using a locally written analysis. The data obtained from three replicate titrations and three replicate ligand dilutions in identical conditions were averaged. The averaged heats of the ligand dilution were then subtracted from the average heats of the titration to give pure averaged binding data [26]. As the change in heat associated with each injection is the slope of the cumulative heat generated, the heats were added to get the cumulative heat Q as a function of total volume of ligand added. This integration turns the ITC data into an NMR-shaped saturation curve so that it can be analysed using [23]:

4 $$\frac{\left[PL\right]}{2n\;{\left[P\right]}_{i\;}{\left[P\right]}_{0}}=\left(n\;{\left[P\right]}_{i}+{\left[L\right]}_{i}+K_{d}\right)-\sqrt{{\left\{n\;{\left[P\right]}_{i}+{\left[L\right]}_{i}+K_{d}\right\}}^{2}-4{\left[L\right]}_{i}\;{\left[P\right]}_{i}}$$

5 $$Q=\Delta H_{\max}\cdot V_{0}\cdot n\;\left[PL\right]$$

Total protein concentration and total ligand concentration after each injection interval were calculated using a model similar to that apparently used by the Microcal software, and described [27] for use with overloaded ITC cells (i.e. typical conditions where the initial volume is already greater than the volume of the sample cell) and assumes that added ligand is mixed within the sample cell but not outside it, where solution is simply displaced from the sample cell without mixing:

6 $${\left[P\right]}_{i}={\left[P\right]}_{0}\;exp\;\left(\frac{-v_{i}}{v_{c}}\right)$$

7 $${\left[L\right]}_{i}={\left[L\right]}_{0}\left(1-exp\left(\frac{-v_{i}}{v_{c}}\right)\right)$$

where *v*_c_ is the calibrated sample cell volume, [*P*]_0_ is the starting protein concentration and [*L*]_0_ is the starting ligand concentration within the syringe. The [*L*]_i_ calculated in this way was converted into monomeric EGCG concentration using the modified isodesmic model. The corrected monomer concentration is no more than 10% different as a consequence of self-association even at the highest concentrations used, so the effect of self-association is small. Any potential changes in self-association constant resulting from the different phosphate concentrations in the NMR and ITC titrations are therefore insignificant. For two independent sets of binding sites, the cumulative heats from both binding events were added.

## Results

### General remarks

It was considered important to have solution conditions as close to physiological (blood serum) as possible. However, this would entail pH buffering using bicarbonate. Since the ITC experiments require extensive degassing, this would adversely affect the pH of the solution. We therefore used physiological metal ion concentrations but phosphate as a pH buffer. Phosphate has a low ionization enthalpy, making it suitable for ITC, and is not present in ^1^H NMR spectra, making it also suitable for NMR.

**Figure 1:**
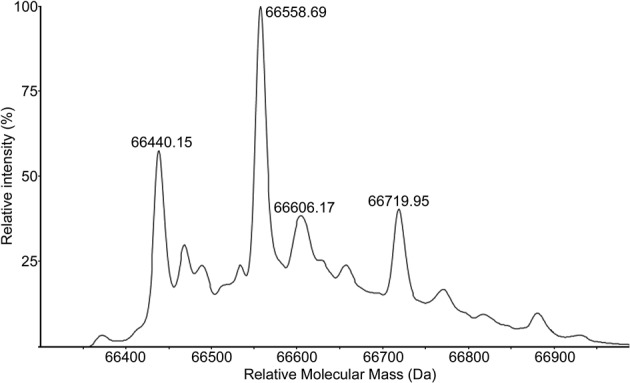
HPLC-ESI-MS spectrum of HSA deconvoluted using manufacturer’s software.

HSA is a mixture of different isoforms and contains some post-translational modifications (PTM). *In vivo*, it is typically bound to a range of lipophilic molecules. We therefore used fatty acid free HSA purified from blood (rather than recombinant protein). SDS/PAGE showed the protein to run as a single band. However, electrospray MS (Figure 1) showed that in addition to the molecular ion (expected: 66443.8 Da), there are a number of other species present, of which the most intense is at a mass 118.5 higher and is likely to be due to N-homocysteinylation (117.2 Da). This is a known PTM that has been reported to reduce the binding of HSA to EGCG by 32% [28].

### EGCG self-association

**Figure 2:**
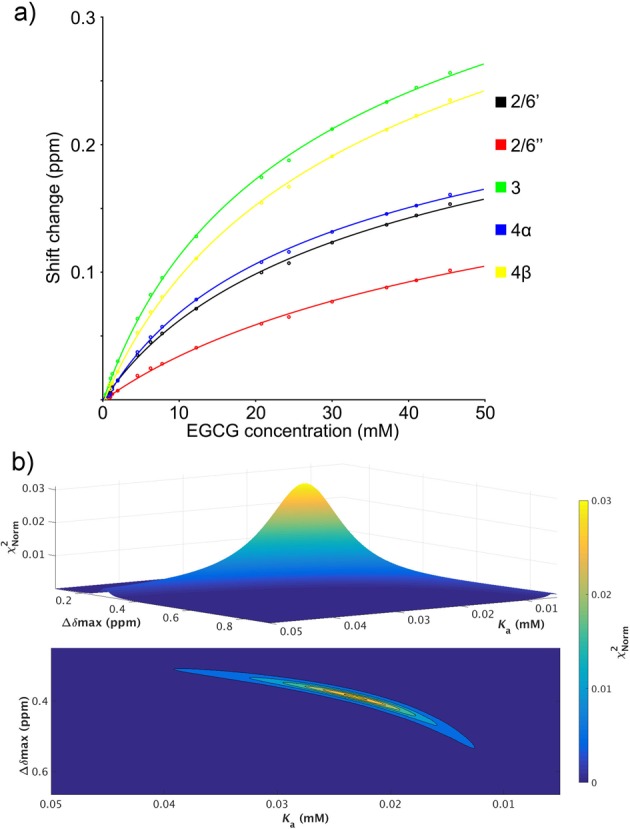
Fitting of the self-association constant for EGCG. (**a**) Chemical shift changes of EGCG as a function of EGCG concentration. (**b**) Plot showing how χ^2^ varies for different values of *K*_a_ and *Δδ*_max_, for the 2′/6′ protons. For ease of viewing, χ^2^ values were converted into a normalized $\chi_{\text{norm}}^{2}=1/(\chi^{2}+1)$. The grid search was carried out automatically using a python script and the results are displayed using Matlab with the surface command and displayed as a 3D plot and a 2D contour plot.

A self-association constant for EGCG has been measured before [29,30], but not under the same solution conditions as used here. We therefore measured chemical shifts for EGCG as a function of concentration (Figure 2a). EGCG assignments were taken from [31]. Chemical shifts were fitted to both the isodesmic and the modified isodesmic models. The modified isodesmic model gave χ^2^ values approximately 25% smaller than the isodesmic model, and is therefore preferred. The fitted values of *K*_a_ and* Δδ*_max_ are interdependent, as expected. In order to check the quality of the fitted values, a grid search was carried out, calculating χ^2^ over a wide range of *K*_a_ and *Δδ*_max_ pairs. The results (Figure 2b, Supplementary Table S1) show that there is a clearly defined optimum, with a *K*_a_ of 50.4 mM.

### NMR studies of EGCG binding

**Figure 3:**
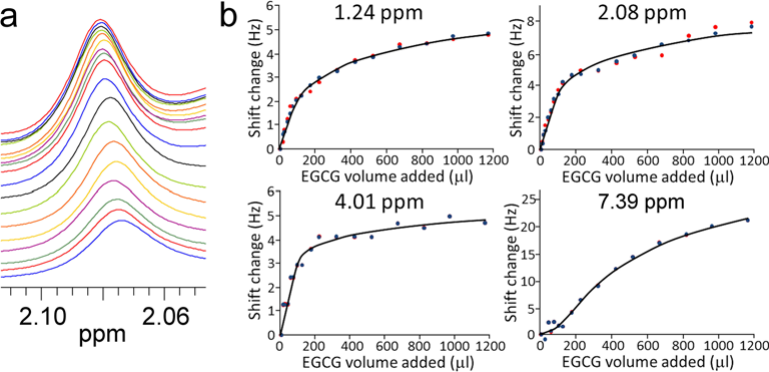
Examples of NMR chemical shift changes observed in HSA on titration with EGCG. (**a**) NMR data for the signal at 2.08 ppm. The first titration point (free HSA) is at the top. (**b**) Shift values for four representative peaks. The original chemical shift data are in red circles, and the SVD-processed data in blue. The binding saturation curves using the best fitted *n* and *K*_d_ are shown in black.

EGCG was titrated into HSA, and the resulting changes in HSA chemical shifts were measured. NMR experiments were conducted in 100% D_2_O in order to exchange out HN protons and resolve aromatic signals better, and a strong resolution enhancement was applied to the data to make peaks sharp enough to measure accurately. The shift changes are small, being in most cases smaller than 20 Hz, making them challenging to measure. Because of the high molecular weight of HSA (roughly 66 kDa), most of the peaks observed are likely to be made up of several individual protons. Three patterns of shifts were observed. In the first group, many peaks had no measurable change of shift. In the second group, shifts changed, but the shift changes bore a clear relationship to the pH and did not have a relationship with EGCG concentration. All these peaks were sharp and in the aromatic region and are likely to come from histidine ring protons. A third group changed with addition of EGCG in patterns resembling standard saturation curves. A selection of these peaks is presented in Figure 3. Approximately 15 peaks had measurable data, and many more regions showed EGCG-dependent shifts that could not be measured accurately, implying that EGCG affects a large number of protons, and therefore that several EGCG molecules bind simultaneously. The fact that a large number of signals show no change in chemical shift strongly implies that even high concentrations of EGCG caused no appreciable structure change, in agreement with [12] but not [8].

Inspection of the data (Figure 3) shows that there are two binding events: a strong binding that is almost complete by 150 μl, and a much weaker binding event. The strong binding event in particular has variable magnitude of effect on chemical shift for different signals, suggesting that it is quite a localized event. One equivalent of EGCG corresponds to an addition of 51 μl, and a simple visual inspection shows that the rollover in the curve occurs at around 100 μl, suggesting that the strong binding corresponds to approximately two molecules of EGCG bound.

We fitted the data to the standard multiple binding model of (eqn 3). Attempts to fit all or part of the data to single binding events gave poor fits and inconsistent results. We therefore fitted the data to a sum of two independent multiple binding events: initially iteratively by eye, and subsequently using the Solver function of Excel. Varying the strong binding affinity *K*_d1_ affects mainly the rollover of the curves at approximately 100 μl, most clearly seen in the peak at 4.01 ppm. The fitted *K*_d1_ is therefore very sensitive to the values of the data points here and insensitive to the rest. Fitting to different signals gave an average *K*_d1_ of 18 ± 8 μM, with *n*_1_ (the number of independent sites) as 1.72 ± 0.26. The fitted value of *K*_d2_ is determined by the overall distribution of the later points in the titration, and fitting gives a rather poorly defined *K*_d2_ of 20 mM or weaker.

In an attempt to improve the quality of the fitting, we processed the raw data using singular value decomposition (SVD). This is a statistical technique related to Principal Component Analysis, and is used extensively in signal processing. It is available in a number of software packages, including Matlab™, widely used in engineering applications. Briefly, a p × q dataset **D** consisting of p rows of data in q conditions (here, p denotes signals and q denotes the titration steps) can be factorized as

8 $$D=UWV^{\text{T}}$$

where **U** is a p × p unitary matrix, **V** is a q × q unitary matrix and **V**^T^ is its transpose, and **W** is a p × q diagonal matrix, whose diagonal elements σ_i_ are real and non-negative. The σ_i_ are the singular values of **D**, and are conventionally presented in descending numerical value. The number n of non-zero σ_i_ defines the rank of the matrix, that is, the number of independent components (i.e. the number of different molecular species whose chemical shifts are required in order to fit the data). Here, we expect the rank to be 3, corresponding to free protein, strongly bound and weakly bound, assuming that the effects on chemical shift of strong binding and weak binding are independent [32]. In practice, none of the σ_i_ is exactly zero, because of noise in the experimental data. However, one can set all the σ_i_ of greater than rank 3 to 0, leaving a reduced **W′** as a 3 × 3 diagonal matrix, and at the same time reduce **U** and **V** to only three columns. The resulting **D**′ matrix, calculated from **D′** = **U′W′V′**^T^, contains much less noise than the original **D**. SVD is thus very useful for removing much of the noise from the data, therefore improving subsequent fitting of the data, and is reported to be the least biased way of extracting the meaningful data from an original overdetermined set containing experimental noise [33]. This procedure was therefore applied to the raw data, resulting in reduced noise (compare red and blue points in Figure 3).

On fitting the data processed using SVD, we obtained *K*_d1_ =19 ± 12 μM and *n*_1_=1.8 ± 0.18, i.e. a very similar estimate of *K*_d1_ to that found using the raw data but a slightly larger and better defined estimate for *n*_1_. Fitting to the weaker binding showed that *K*_d2_ is greater than 10 mM but that (in a similar way to the self-association data) *K*_d2_ and Δδ_max2_ are correlated: increasing *K*_d2_ gives calculated shifts that can be fitted equally well by a larger Δδ_max2_ (Supplementary Figure S1). We therefore placed limits on the fitted *K*_d2_ by considering the range of chemical shift changes expected for the multiple binding of a polyphenol to a protein surface. A number of publications on tannins binding to proteins suggest that Δδ_max_ values of larger than 0.1 ppm (here 80 Hz) are rare. This is particularly true in geometrically poorly defined complexes, where the dynamic structure of the complex produces averaged shift changes which are smaller in magnitude [24]. We therefore expect typical Δδ_max2_ of less than 80 Hz, implying a *K*_d2_ in the range 20–60 mM. The fitted value of *n*_2_ corresponding to this *K*_d_ is 21 ± 16.

### ITC studies of EGCG binding

**Figure 4:**
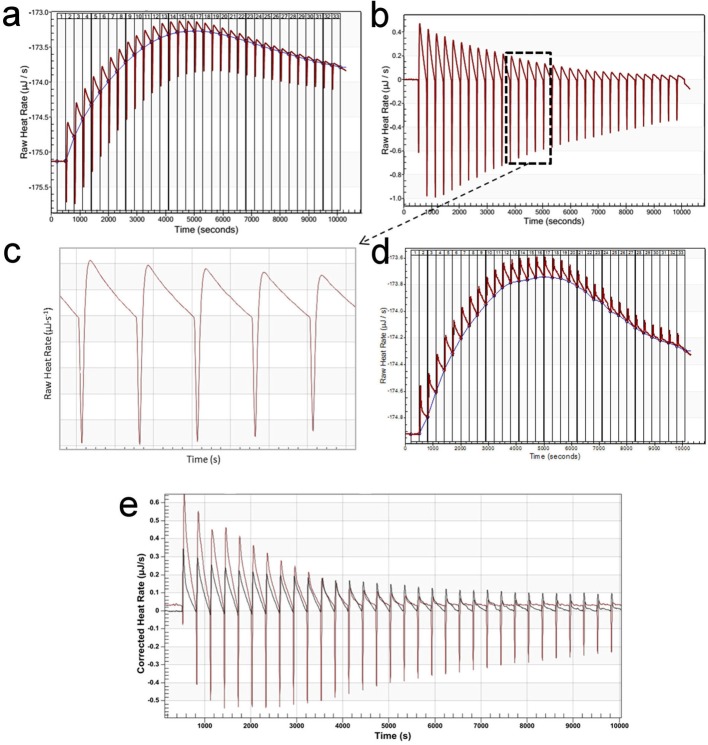
Control ITC data: titration of EGCG into phosphate buffer. (**a**) Titration of 5 mM EGCG into buffer, 37°C, 300 s between injections. Exothermic is up and endothermic is down. There is an initial rapid endothermic change, followed by a slower exothermic change, which leads to a rise in the baseline. The baseline change only stabilizes once the exothermic change becomes small. (**b**) Same as (a) after baseline correction. (**c**) Expansion of (b). (**d**) Same as (a) but using 2.5 mM EGCG. (**e**) Baseline-corrected titrations using 5 mM EGCG (red) and 2.5 mM EGCG (black). Using 2.5 mM EGCG, there is no rapid endothermic change.

Control titrations of EGCG into phosphate buffer (Figure 4a–c) showed a rapid endothermic effect and a slower exothermic effect, which led to a drift in the baseline. Similar behavior was observed previously during ITC titrations of EGCG into a salivary proline-rich peptide [34]. The authors concluded that the endothermic effect is due to a combination of ligand dilution and dissociation of EGCG aggregates. This explanation seems likely, considering the 50 mM dissociation constant observed here, and the fact that 2.5 mM EGCG has essentially no endothermic effect (Figure 4d). The exothermic effect is less easily explained. Its magnitude is roughly proportional to the concentration of EGCG added (Figure 4e) and it happens both in the presence and in the absence of protein. Experiments at 20°C showed similar effects (results not shown) implying that this is not related to specific heat capacity. It is likely to be due to a slow coating of EGCG on to the sample cell or syringe.

**Figure 5:**
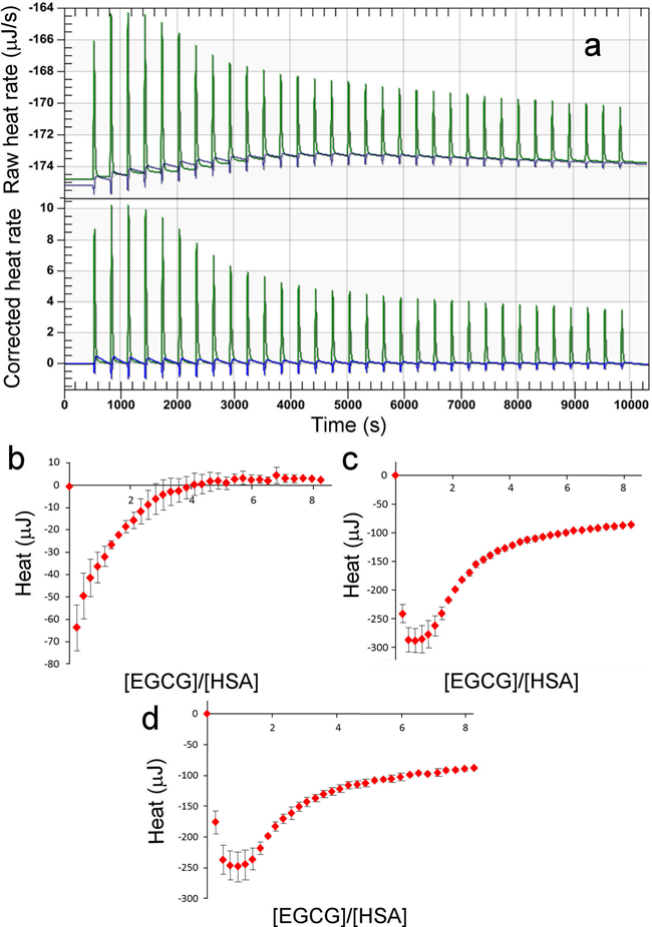
ITC titration of EGCG into HSA. (**a**) Titration of 5 mM EGCG into 200 μM HSA (green) compared with EGCG into buffer (blue). Top is the raw data and bottom after baseline correction. (**b**) Heats of titration of EGCG into buffer, shown as mean and S.D. (*n*=3). (**c**) Heats of titration of EGCG into HSA, shown as mean and S.D. (*n*=3). (**d**) Difference between c and b, which corresponds to the pure heat of interaction.

Titration of EGCG into HSA gave much larger changes in enthalpy (Figure 5). Titrations generated a very similar baseline drift to that seen in the absence of protein, providing confidence that the heat changes after baseline correction are genuinely due to binding. In order to obtain reliable data, titrations were repeated three times, and the difference between protein and buffer was calculated, to produce pure heats of binding to protein. At low EGCG concentrations, the data resemble typical ITC profiles, but the heats of binding tail off very slowly as EGCG concentration is increased, indicating two interactions, one much weaker than the other.

Fitting these results to an independent binding sites model using the NanoAnalyze software supplied with the calorimeter gave a poor fit to the data. The software is unable to deal with the tail at high [EGCG]. One can either ignore titration data beyond a cutoff [EGCG] or subtract a constant heat from the data to give an artificial baseline. Both procedures are unsatisfactory. We therefore adopted an approach similar to that used by [35]. Heats of titration were added to give a cumulative heat at each titration point. These curves follow (eqn 4), which has the same form as NMR chemical shift titrations (eqn 3)). A further benefit of carrying out an independent fitting is that we were able to correct the EGCG concentration for the small proportion of self-associated EGCG at higher concentration. The resultant data fit well to two sets of independent sites model, and give *K*_d1_ =22 ± 4 μM and *n*_1_=2.5 ± 0.03, and *K*_d2_ =21.6 ± 3.5 mM and *n*_2_=9 ± 1.

## Discussion

ITC and NMR give consistent results and the combination of the two methods gives a much greater degree of confidence in the result, as well as a more complete description. Significantly, both methods show that EGCG binds with two very different sets of affinities. There is a strong binding event with a *K*_d_ of approximately 20 μM, and a weak binding with a *K*_d_ approximately 1000 times weaker. The important result shown here is that the strong binding involves the binding of two molecules of EGCG per HSA, while the weak binding involves approximately 12. Thus, the number of EGCG molecules bound under physiological conditions is much greater than found previously. We note that NMR is able to give a robust estimate for both binding events, despite the weak binding, small shift changes and large size of the protein. Data processing with SVD was useful in improving the quality of the fitting. On comparing our results with previous determinations, it is clear that fluorescence quenching is somewhat misleading, because it only characterizes binding at Sudlow site I, where the single tryptophan of HSA is located and therefore only gives *n*=1 [8–10]. This is to be expected, but is not clear from previous reports. Our measurements give an estimated combined fit of *K*_d1_ =21 ± 4 μM and *n*_1_=2.2 ± 0.3, and *K*_d2_ =30 ± 10 mM and *n*_2_=12 ± 5. *K*_d1_ is weaker than any previous value. We have strong confidence in the value of *n*_1_=2, i.e. strong binding at approximately two equivalent sites, presumably Sudlow sites I and II, which are typically found as the main binding sites for hydrophobic ligands. One would expect different affinities at the two sites. Our measurements do not allow us to distinguish between the two affinities. The affinities for the two sites thus appear to be similar in magnitude, and the experimentally determined value of 21 µM is an average of the values for the two individual sites. The general picture of a strong and specific binding at a small number of sites, plus a much weaker general surface binding, is not unexpected [17,35]. The large value of* n*_2_ is much higher than previous estimates for related systems, though consistent with the wide range of binding sites for polyphenols identified in previous studies [36]. Previous studies of binding to both HSA and BSA have suggested that catechins bind either at Sudlow site I or at both sites I and II. Our results demonstrate two equivalent strong sites and therefore support equivalent binding at both Sudlow sites. Previous studies have not observed the weak binding events. In many cases, this is because titrations were not continued to high concentration; in some cases, the spectral changes at high concentration were observed but ignored.

During the NMR titration, small chemical shift changes were observed that were clearly a consequence of pH changes, despite the protein and ligand being in the same buffer at the same pH. This is presumably due to displacement of protein-bound protons by the incoming ligand, a phenomenon widely used to measure metal-binding affinities [37]. The energetic changes that accompany the proton displacement form a part of the overall observed affinity.

We also attempted to measure the binding affinity of EGCG to HSA using Microscale Thermophoresis (MST). This proved unsuccessful, using either red or blue dyes supplied by NanoTemper Technologies GmbH, because the EGCG strongly quenched the dye fluorescence (Supplementary Figure S2). This result supports the surface binding of EGCG at multiple locations.

It has been proposed that binding to albumin, and particularly to the hydrophobic Sudlow pockets, stabilizes EGCG and prevents degradation and metabolism. The peak concentration of EGCG reached in humans after drinking a cup of green tea is approximately 0.2 μM, though it may be higher when drinking several cups a day [3]. The concentration of free ligand in the presence of *n* equivalent binding sites can be calculated from the standard equation:

9 $$Fraction\;of\;protein\;sites\;bound=\frac{\left[PL\right]}{{\left[P\right]}_{i}}=\frac{n\;\left[L\right]}{K_{d}+\left[L\right]}\approx \frac{n\;\left[L\right]}{K_{d}}$$

where [L] and [PL] are the concentrations of free ligand and protein-bound ligand. Given a time-averaged EGCG concentration of 0.1 μM, a concentration of HSA of 600 μM, *K*_d1_ =21 μM and *n*_1_=2, we calculate that the fraction of HSA-binding sites occupied by EGCG is less than 0.01%, implying that consumption of EGCG has no effect on the ability of HSA to bind and transport other molecules. More significantly, these values imply that only 1.7% of the EGCG is free, the rest being bound to HSA. Previous estimates of *K*_d_ and *n* have come out at approximately 15 μM and 1 respectively [9,10]. Use of these values in the equation results in a higher fraction of EGCG estimiated to be free, namely 2.4%. The weak binding measured here is much weaker but has a larger number of sites, and contributes to reduce the concentration of free EGCG by a further 19%, giving a proportion of free EGCG in serum of only 1.4%. Thus, almost all the EGCG in serum is bound to HSA (and more than calculated from earlier studies, where *n*_1_=1), almost all in the hydrophobic Sudlow pockets. If binding to HSA protects EGCG against degradation, then our results suggest how this works. It is likely that almost all of the remaining ‘unbound’ EGCG is bound to other proteins, further reducing the amount of free EGCG.

Weaker binding normally implies faster off-rates, because of the inverse relationship between *K*_d_ and *k*_off_ [24]. Our result therefore implies that EGCG is likely to be released rapidly from HSA, at a rate of at least 100 s^−1^. Thus, release of EGCG to tissue is likely to be fast and not rate-limiting.

This result provides an explanation for the wide tissue distribution [3] and slow metabolism [38–40] of EGCG *in vivo* [8]. It further suggests that *in vitro* assays of EGCG activity would be more physiologically realistic if carried out in the presence of albumin.

## Abbreviations

EGCG: epigallocatechin gallate
HSA: human serum albumin
ITC: isothermal titration calorimetry
*K*_a_: self-dissociation constant
*K*_d_: dissociation constant
MST: microscale thermophoresis
PTM: post-translational modification
SVD: singular value decomposition
TSP: 3-(trimethylsilyl)-2,2′,3,3′-d_4_ propionate
